# Identification of miR-93 as a suitable miR for normalizing miRNA in plasma of tuberculosis patients

**DOI:** 10.1111/jcmm.12535

**Published:** 2015-03-08

**Authors:** Simone E Barry, Brian Chan, Magda Ellis, YuRong Yang, Marshall L Plit, Guangyu Guan, Xiaolin Wang, Warwick J Britton, Bernadette M Saunders

**Affiliations:** a Centenary InstituteNewtown, NSW, Australia; b Sydney Medical School, University of SydneySydney, NSW, Australia; c Department of Thoracic Medicine St Vincent’s HospitalDarlinghurst, NSW, Australia; d Ningxia Medical UniversityYinchuan, Ningxia, China; e QIMR Berghofer Medical Research InstituteBrisbane, QLD, Australia; f Ningxia Centre for Disease Control and PreventionYinchuan, Ningxia, China; g Infectious Disease Hospital of NingxiaYinchuan, China

**Keywords:** microRNA, tuberculosis, respiratory disease, plasma, normalisation

## Abstract

Tuberculosis (TB) remains a major public health issue. New tests to aid diagnoses and monitor the response to therapy are urgently required. There is growing interest in the use of microRNA (miRNA) profiles as diagnostic, prognostic or predictive markers in a range of clinical and infectious diseases, including *Mycobacterium tuberculosis* infection, however, challenges exist to accurately normalise miRNA levels in cohorts. This study examined the appropriateness of 12 miRs and RNU6B to normalise circulating plasma miRNA levels in individuals with active TB from 2 different geographical and ethnic regions. Twelve miRs (let-7, miR-16, miR-22, miR-26, miR-93, miR-103, miR-191, miR-192, miR-221, miR-423, miR-425 and miR-451) and RNU6B were selected based on their reported production by lung cells, expression in blood and previous use as a reference miRNA. Expression levels were analysed in the plasma of newly diagnosed TB patients from Australia and China compared with individuals with latent TB infection and healthy volunteers. Analysis with both geNorm and NormFinder software identified miR-93 as the most suitable reference miR in both cohorts, either when analysed separately or collectively. Interestingly, there were large variations in the expression levels of some miRs, in particular miR-192 and let-7, between the two cohorts, independent of disease status. These data identify miR-93 is a suitable reference miR for normalizing miRNA levels in TB patients, and highlight how environmental, and possibly ethnic, factors influence miRNA expression levels, demonstrating the necessity of assessing the suitability of reference miRs within the study population.

## Introduction

Tuberculosis (TB) is an infectious disease caused by the bacillus, *Mycobacterium tuberculosis* that normally affects the lungs. It is estimated that one-third of the world’s population is infected with *M. tuberculosis*. Of those infected however, only about 10% will ever develop active disease 1 whereas the rest remain in a latent state. It is estimated that TB results in the death of about 1.5 million individuals annually 2. Diagnosis of TB is made most commonly by sputum smear microscopy. Mycobacterial culture remains the gold standard for TB diagnosis, with rapid molecular tests such as genXpert®, also employed where available 3. However, lengthy delays in diagnosis are an ongoing problem in TB management. Therapy for TB disease requires a minimum of 6 months on multiple antibiotics. Monitoring a patient’s response to infection to identify non-responders, which may be indicative of poor compliance or drug resistance, is another important requirement for managing TB. Currently, conversion into sputum negative culture at 2 months is the gold standard marker of successful treatment, but this means it is often 3 months or more into therapy before non-responders are identified, during which time they remain infectious. New biomarkers that aid diagnosis and identify treatment non-responders early, quickly and cheaply would greatly assist in reducing the burden of TB infection 1.

miRNAs are single-stranded RNA molecules of 21–23 nucleotides in length that play an important role in post-translational regulation of gene expression through targeting mRNA 4. They circulate in a stable, cell-free form and are increased in levels in many disease processes such as malignancy, schizophrenia, heart failure and sepsis 5–7. Their use as a biomarker to aid diagnosis and predict response to therapy is of growing interest. They may also represent a novel therapeutic target to modify the host response.

Normalization of miRNA levels is critically important to enable correction of inter-sample variation and differing reaction efficiencies. Commonly, RNU6B has been used in many cell and tissue studies, but other studies have shown that RNU6B is not stably expressed in sera 8. To date, there are no published studies that systematically evaluate reference miRs for normalizing plasma-derived miRNAs in the setting of TB 9,10 or in different ethnic and geographical groups to determine if these factors influence miR expression.

The aim of this study was to identify suitable and stable reference miRs for normalization of miRNA levels in patients with active TB, compared with individuals with latent tuberculosis infection (LTBI) and healthy controls and to examine if geographical or ethnic differences influence miRNA levels in TB patients in Australia and the Ningxia Hui Autonomous Region of north-west China 11.

## Materials and methods

### Ethics statement

This study was undertaken with the approval of the Ethics Review Committees at St Vincent’s Hospital, Sydney (Protocol No. X11-0141 and HREC/11/RPAH/201), The University of Sydney (Protocol No. 2012/1076) and The Ningxia Medical University (approval date 6/6/2013). All participants provided written informed consent.

### Australian cohort

We invited suitable consecutive consenting patients presenting to the TB clinic at St Vincent’s Hospital to participate in the study. Enrolment for the study ceased, once we had reached the required enrolment quota. Blood was collected from 12 patients with culture-proven pulmonary TB, 12 with LTBI (based on a Tuberculin skin test of greater than or equal to 15 mm, a lack of symptoms and a normal chest radiograph) and 12 healthy controls with no exposure to TB. Of peripheral blood, 10 ml was collected into EDTA tubes and processed within 3 hrs.

### The Peoples Republic of China cohort

Blood was collected from 12 patients with either culture-proven TB or TB diagnosed on clinical and radiological grounds and 12 matched healthy controls. Of peripheral blood, 10 ml was collected into ETDA tubes and processed within 3 hrs. All patients recruited from China were born in Peoples Republic of China (PRC) and classified themselves as Hui ethnicity (Table1).

**Table 1:** Baseline characteristic of subjects by country of birth and disease state

Cohort	Australian	Australian	Australian	PRC	PRC
Clinical pathological variable	TB *n* = 12	LTBI *n* = 12	Healthy control (*n* = 12)	TB *n* = 12	Healthy control (*n* = 12)
Median age, years (range)	36 (20–60)	34 (18–59)	44 (27–76)	45 (16–76)	39 (16–68)
Male (%)	4 (33.3)	6 (50)	6 (50)	11 (92%)	7 (58)
Place of birth*	South Asia (*n* = 2)	South East Asia (*n* = 3)	PRC†	PRC†
	East Asia (*n* = 6)	Southern Africa (*n* = 1)			East Asia (*n* = 1)	Western Europe (*n* = 1)
	South Eastern Asia (*n* = 3)	East Africa (*n* = 1)				
		Eastern Europe (*n* = 1)				
Oceania(*n* = 10)	Oceania (*n* = 6)	Oceania (*n* = 1)

* Regions as classified by the WHO.

† All PRC subjects of HUI ethnicity.

### RNA extraction and reverse transcription

Blood was centrifuged at 1500 × g for 10 min. at RT, and the upper aqueous phase removed into 2 ml eppendorf tubes and centrifuged for 3 min. at maximum speed. Plasma was removed and stored at −80°C until required. Chinese samples were shipped to Australia at temperatures below −60°C and stored at −80°C until required.

The RNA in 500 μl of plasma was extracted with 1 ml of TRIzol LS (Life Technologies, Melbourne, VIC, Australia) as per manufacturer’s instructions with one minor modification of an overnight incubation with isopropanol before samples were resuspended in 20 μl of H_2_O. RNA was measured using a NanoDrop 2000 spectrophotometer (Thermo Scientific, Wilmington, DE, USA). Reverse transcription was carried out using all-in-one miRNA first cDNA synthesis kit (Gencopoeia, Rockville, MD, USA) according to the manufacturers’ instructions and undertaken on a thermal cycler with the following parameters; 37°C for 60 min. and 85°C for 5 min. The resulting product was stored directly at −20°C.

### qPCR to detect miRNAs

qPCR was performed in 10 μl reactions using the all-in-one qPCR kit (Gencopoeia), as per the manufacturer’s instructions and analysed on an ABI 7500. The PCR reactions were initiated with 10 min. incubation at 95°C followed by 40 cycles of 95°C for 10 sec. and 60°C for 10 sec., with melting curve analysis performed to verify the amplification.

### Data analysis

Data were analysed using SDS Relative Quantification Software Version 2.3. Endogenous controls must be consistently expressed thus only the miRNAs detected in all 60 samples were selected for further analysis using the Normfinder and geNorm software packages (GenEx Version 5, Multi D, Göteborg, Sweden). Normfinder and geNorm reference gene validation software was used to identify the most stably expressed miRs.

### Statistical analysis

The difference in miR expression was analysed by Student’s *t*-tests for comparison of two groups and Wilcoxon rank-sum test for multi-group comparisons, *P*-values below 0.05 were considered significant.

## Results

The clinical characteristics of study participants are listed in Table1. The Australian patients with TB were mainly born in Asia (91%), with one-third being male. Those with LTBI were born in a variety of regions with 50% from the Oceania region. In the Australian healthy control group, the vast majority were born in Australia (83%). All subjects in the PRC cohort were born in PRC and identified themselves as Hui in ethnicity. They were 92% male with an average age of 42 years. The extent of the TB disease as classified radiologically was generally less advanced in the Australian group compared with the PRC cohort suggestive of later presentation and/or diagnosis and possible concurrent chronic lung disease in the PRC cohort.

**Figure 1:**
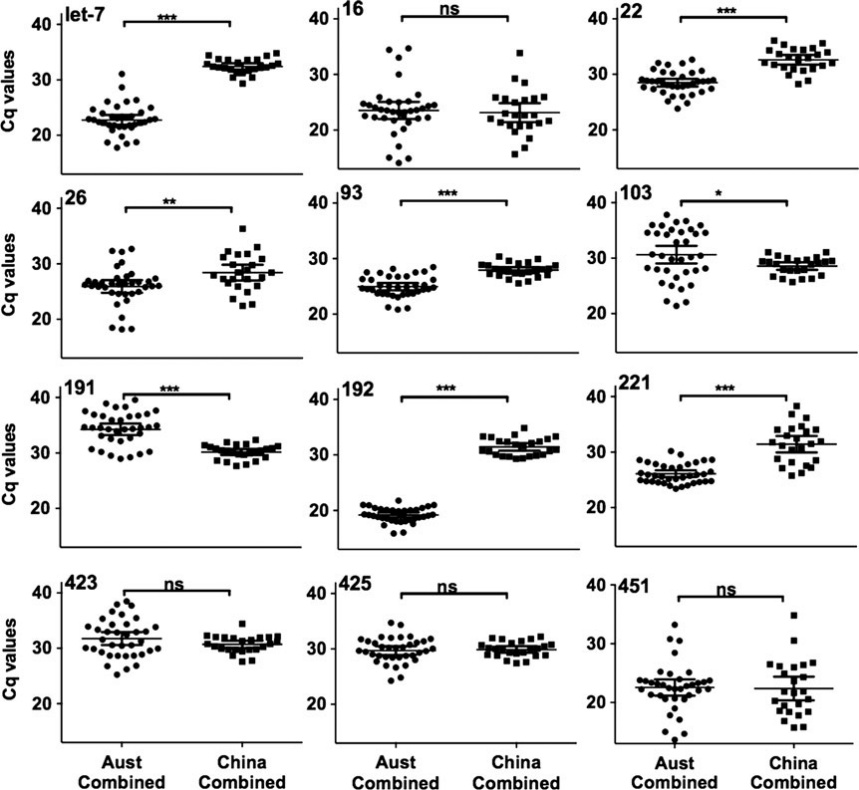
Expression levels of candidate reference miRNAs by cohort. Expression levels of the 12 candidate reference miRNAs in the combined Australian cohort (*n* = 36) and the PRC cohort (*n* = 24) were determined by qPCR. Values are given as the quantification cycle (Cq mean of duplicate sample). Significant differences were calculated by Wilcoxin rank-sum test. *0.01 to <0.05, **0.001 to <0.01, ***<0.001, ns = not significant.

The Cq values for the 13 candidate miRs were measured for all 60 individuals, with Cq thresholds ranging from 13 to 39 (Table2). RNU6 was poorly expressed with Cq values often above the threshold of detection and was therefore excluded from further analysis. The expression levels of some miRs varied markedly between the two populations, independent of disease status (Fig.1). Two miRs (miR-192 and let-7a) were highly variable between the cohorts with differences in 7–9 cycles (128- to 512-fold) between average values for the cohorts, while the level of expression of four other miRs (miR-26, -16, -423, -425) were within one cycle between the PRC and Australian cohorts (Table2). By stratifying groups based on location, we found statistically significant differences in many of miRs examined that were influenced by cohort not disease state.

**Table 2:** Expression levels of candidate reference genes in the Australian and PRC cohort, independent of disease status

miRNA	Cq mean (range) Australian cohort *n* = 24	Cq mean (range) PRC cohort *n* = 36	Cq mean variability between cohorts
192	22.14 (15.87–33.31)	31.46 (29.33–34.84)	9.31
22	29.59 (23.81–35.59)	32.62 (28.23–36.08)	3.03
221	26.79 (23.41–32.95)	31.42 (25.77–38.31)	4.64
26	25.90 (18.18–32.68)	28.41 (22.42–36.34)	2.50
451	21.56 (13.66–33.21)	22.37 (15.73–34.82)	0.81
93	25.64 (20.82–29.13)	27.93 (25.53–30.35)	2.29
let 7	25.38 (17.77–34.41)	32.45 (29.32–34.79)	7.06
16	22.69 (14.07–34.70)	23.13 (15.67–33.86)	0.45
103	30.20 (21.40–37.81	28.58 (25.70–31.07)	−1.62
191	33.36 (28.96–39.57)	30.20 (27.66–32.35)	−3.16
423	31.53 (25.25–38.50)	30.72 (27.58–34.43)	−0.81
425	29.76 (24.26–34.71)	29.92 (27.41–32.19)	0.15

Values are given as mean and range in brackets of the quantification of cycle (Cq) values of candidate reference genes by cohort.

### Determination of reference miRNA expression stability using geNorm and Normfinder

geNorm 12 and Normfinder 13 software were utilized to examine the stability of candidate reference miRs from the plasma of all groups from both cohorts. Both geNorm and Normfinder assume equal variance of the candidate miRs between the populations. Therefore, a Levene’s test of variance was carried out on all miRs from the respective cohorts to determine if there was significant variance between the groups 14. miRs with *P*-values below 0.05 were considered significantly different, and were excluded from further the analysis. In the PRC cohort, four miRs were excluded (miR-16, -26, -221 and -451), while none were excluded from the Australian cohort, and overall when all samples were examined collectively, four miRs were excluded from subsequent analysis (miR-26, -192, -221 and -451).

For Normfinder, the maximum expression of each candidate reference miR (as given by lowest Cq value) was used as a control and set to 1. Relative expression levels where then calculated from Cq values using the formula: 2^−ΔCq^, in which ΔCq is equal to the corresponding Cq value minus the minimum Cq value. For geNorm analysis, data files containing the mean Cq values as determined by SDS Relative Quantification Software were imported directly into GenEx software for analysis. geNorm generates an M value based on the average standard deviation of the ratio of the pairwise normalization miRs accompanied by stepwise exclusion of the least stable miR. The lower the M value, the more stable the miR.

### miR-93 and miR-425 are the most stable reference miRNA in the PRC cohort

**Figure 2:**
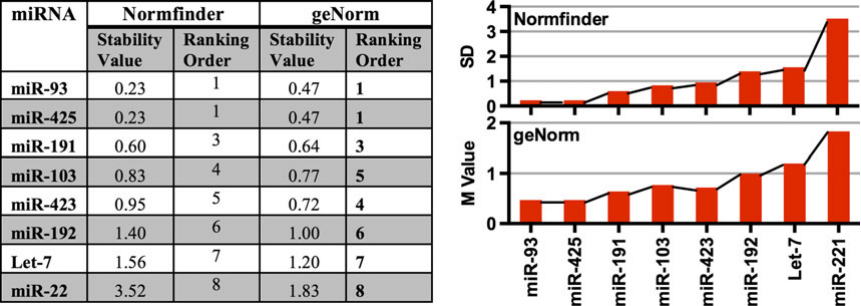
Stability values of candidate reference genes for the PRC cohort as determined by Normfinder and geNorm. Expression levels of 12 miRNAs in the plasma of newly diagnosed TB patients (*n* = 12) and healthy controls (*n* = 12) were subject to a Levene’s test to determine there was significant variance between groups. miRs -16, -26, -221 and -451 displayed significant variance between groups and were removed from subsequent analysis. The remaining 8 miRNAs were ranked by Normfinder and geNorm. Greater expression stability is indicated by a lower stability value (M). Low standard deviation (SD) values indicate stable gene expression (Normfinder).

Analysis of the PRC cohort using both Normfinder and geNorm identified the expression of miR-93 and miR-425 to be the most stably expressed miRs (Fig.2).

### miRs-93, -221, -22 and let-7 are the most stable reference miRNAs in the Australian cohort

**Figure 3:**
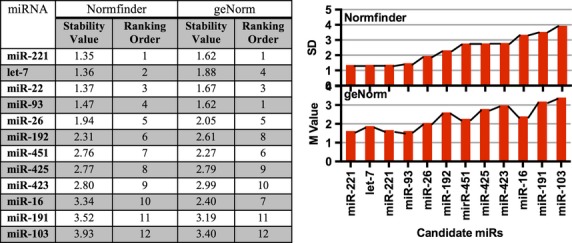
Stability values of candidate reference genes for Australian cohort as determined by Normfinder and geNorm. Expression levels of 12 miRNAs in the plasma of newly diagnosed TB patients (*n* = 12) and healthy and latent controls (*n* = 24) were subject to a Levene’s test which determined there was no significant variance between any of the groups. The 12 miRNAs were then ranked by Normfinder and geNorm. Greater expression stability is indicated by a lower stability value (M) (geNorm). Low standard deviation (SD) values indicate stable gene expression (Normfinder).

geNorm and Normfinder analysis of the Australian cohort identified a panel of four miRs, including miR-93, as suitable reference miRs in this cohort. The rankings of the candidates as determined by are shown in Figure3. The top five miRs were the same using both analysis tools with similar M values between the five miR’s, suggesting that either in combination or alone they are suitable normalisers for this cohort. miR-93 was found to be the most stable when utilizing geNorm software, and although it is ranked number 4 according to Normfinder, the stability values differed little between the top 4 candidates.

### miR-93 and miR-22 are the most stable reference miRNAs in a combined cohort analysis

**Figure 4:**
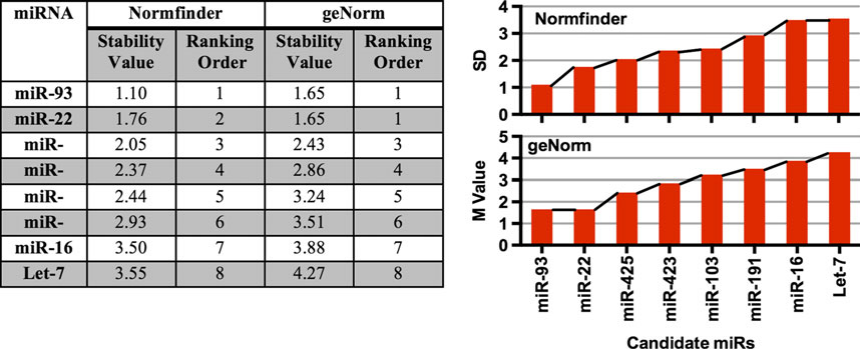
Stability values of candidate reference genes for all participants as determined by Normfinder and geNorm. Expression levels of 12 miRNAs in the plasma of newly diagnosed TB patients in both the Australian and PRC cohort (*n* = 12/group) and healthy and latent controls (*n* = 12/group). A Levene’s test determined there was no significant variance between any of the groups. The 12 miRNAs were then ranked by Normfinder and geNorm. Greater expression stability is indicated by a lower stability value (M).

Examining all patient groups from both cohorts, geNorm and Normfinder identified miR-93 and miR-22 to be the most stable (Fig.4), although the stability values in the combined groups were higher than when each cohort was analysed separately. To explore this in more detail, we examined miR variation across the cohorts.

### Variation in miRNA expression may be cohort rather than disease driven

**Figure 5:**
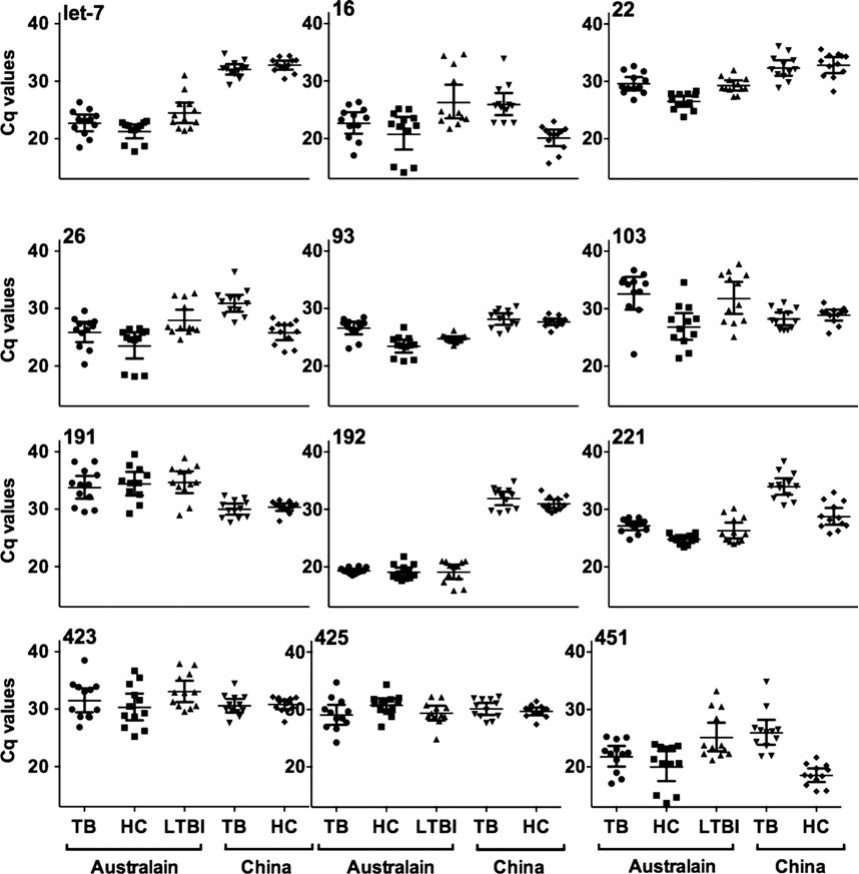
Expression levels of candidate reference gene in healthy controls, latent *Mycobacterium tuberculosis* infection and *M. tuberculosis* disease. Expression levels of the 12 candidate reference miRNAs in the plasma of TB patients, healthy controls and latently infected individuals from Australia and in TB patients and healthy controls from the PRC (*n* = 12/group). Values are given as the cycle threshold (Cq mean of duplicate sample).

The Cq values for the 12 miRs examined in the five groups were plotted by disease state and geographical location (Fig.5). This data suggest that there is variation in miR expression that is independent of disease state, but relates to geographical or potentially ethnic differences between the cohorts. Further studies in larger cohorts will be required to confirm this. Despite these differences in miR expression between the populations, both geNorm and Normfinder analysis identified miR-93 as an appropriate reference miR to normalise miRNA levels in plasma samples from both cohorts independent of disease status.

## Discussion

A growing body of evidence has highlighted the critical regulatory roles that miRNA play to tightly regulate biological processes. miRNAs are commonly found in the blood, often associated with cellular vesicles, such as microparticles or exosomes 15,16. miRNA are stable, and numerous studies have investigated the potential of plasma miRNAs to serve as biomarkers for multiple conditions, such as cancer, stroke and infection 17–20. Normalization of miRNA levels in samples is an essential step in biomarker analysis. This study examined the suitability of 12 miRs to normalise miRNA levels in plasma from TB patients and controls in two geographical and ethnically diverse populations. We found significant variability in miRNA levels across these populations, independent of disease status. Despite these differences, we have identified miR-93 to be a stable, plasma-based miR suitable to normalise miRNA levels across diverse ethnic and geographical populations.

Many of the current miRNA normalization strategies have been adopted from tissue studies where U6 is often used as a reference to normalise miRNA data 21. Recent studies, however, have found that U6 is not stably expressed in plasma 22,23. Our study confirmed these recent findings as U6 was not stably expressed in the plasma of our participants, indeed in over 75% of our samples U6 was not detected within the 40 cycles of the qPCR assay (data not shown). For this reason, U6 was excluded from further analysis as it is not suitable as a reference miRNA for plasma samples. Other studies have used non-human miRNA from *Caenorhabditis elegans* added to samples to control for technical variations, however, it does not control for variation in differences in the efficiency of the reverse transcription 24.

This study examined the expression of miRs previously reported as suitable to normalise miRNA levels in a variety of biological tissues 25–32. The selection of miRs to normalise miRNA is influenced not only by the tissue being examined but also the disease condition being evaluated. miR-16 and miR-451 are commonly used to normalise miRNA levels in plasma samples 30. More recent studies have reported up-regulation of plasma miR-16 and -451 in plasma as a part of the miRNA signature in multiple conditions, including autoimmune thyroid disease 33, experimental sepsis 34 and gastric cancer 35. Another study demonstrated that miRs-16 and -451 are highly expressed in red blood cells so that any haemolysis of blood samples strongly affects their level in plasma 36. We found variable expression of miR-16 and -451 between individuals, such that these miRs did not meet the criteria for inclusion as a stable reference for changes in TB disease in either population.

The development of a blood-based biomarker to identify TB disease and monitor response to therapy would be a major advance for TB diagnostics. Although application of genXpert® has increased the speed of diagnosis, TB diagnosis may still take weeks to months, caused by delays in obtaining appropriate samples, lack of culture facilities and the need for well-trained staff and sophisticated laboratories.

This study has identified significant variability in miR expression in different cohorts and this has important implications for identifying suitable reference miRs, an essential component in accurately analysing qPCR data. While the exact reason for this variation has not been determined, recent reports have shown the miR expression can be influenced by ethnic diversity 37. As has been demonstrated, there is an important need to accurately correct for non-biological variation with stable reference genes that are suitable for the population being studied, otherwise important regulated miRs that may have a role as biomarkers of disease, may be missed. Our data demonstrate the importance of determining the suitability of a reference miR directly in the study population to account for variations in miRNA expression attributable to ethnic and environmental factors.

In general this study found that individuals from the PRC, grouped together more closely than the Australian cohort, whose ethnic background was far more heterogeneous, and included individuals born in nine different countries. In the PRC population, in part due to the tight clustering of the samples, a Levene’s test of variance identified four miRs that were differentially regulated and were therefore excluded from further analysis in that cohort. When the Australian cohort as analysed alone no miRs were excluded after the Levene’s test, but the stability values demonstrate that, compared to the PRC cohort, there was a greater degree of variability in this cohort. geNorm analysis, which indicates that optimal number of miRs that should be used to normalise the samples, found that the best stability values were achieved with analysing four miRs for the Australian cohort, but only four miRs was required to achieve a similar stability value in the PRC cohort. When the two cohorts were combined, a number of miRs were again removed after application of the Levene’s test. The baseline expression of some miRs, most notably miR-192 and Let 7a, differed by 128- to 512-fold between the Australian and PRC cohort. Most of this variation was cohort and not disease driven, suggesting that ethnic and/or environmental factors contribute to baseline miRNA levels in the blood. The increased variability seen in the Australian cohort may also reflect the increased ethnic variation in the participants or other undefined environmental factors. The individuals participating in this study were not screened or asked about other potentially confounding factors. Comorbidities, such as diabetes or concurrent infections, are likely to also influence miRNA expression, and further research is required to evaluate the impact of comorbidity factors on an individual’s miRNA disease signature.

miR-93 has been identified as a suitable a reference miR to normalise miRNA levels in studies of disparate diseases from gastric cancer to major depression disorders 28,30. Other studies in ovarian and breast cancer have found that miR-93 is differentially regulated in cancer tissue and levels may be predictive of disease state 38,39, further highlighting the need to confirm the suitability of selected miRs to normalise miRNA levels depending upon the tissue under investigation, the disease state and potentially the ethnicity of the subject population. In summary, miR-93 is a suitable reference miR for analysing miRNA levels in TB patients, however, ethnic and environmental factors influence miRNA expression in addition to the effect of specific diseases and the suitability of a reference miR should be assessed directly in each study population.
